# The Predictive Processing Model of EMDR

**DOI:** 10.3389/fpsyg.2019.02267

**Published:** 2019-10-04

**Authors:** D. Eric Chamberlin

**Affiliations:** Chamberlin Applied Neuroscience, Glastonbury, CT, United States

**Keywords:** psychological trauma (PTSD), Free Energy Principle, predictive processing, EMDR, memory reconsolidation, physiological mechanism

## Abstract

Eye Movement Desensitization and Reprocessing Therapy (EMDR) is an effective treatment for Post-traumatic Stress Disorder (PTSD). The Adaptive Information Processing Model (AIP) guides the development and practice of EMDR. The AIP postulates inadequately processed memory as the foundation of PTSD pathology. Predictive Processing postulates that the primary function of the brain is prediction that serves to anticipate the next moment of experience in order to resist the dissipative force of entropy thus facilitating continued survival. Memory is the primary substrate of prediction, and is optimized by an ongoing process of precision weighted prediction error minimization that refines prediction by updating the memories on which it is based. The Predictive Processing model of EMDR postulates that EMDR facilitates the predictive processing of traumatic memory by overcoming the bias against exploration and evidence accumulation. The EMDR protocol brings the traumatic memory into an active state of re-experiencing. Defensive responding and/or low sensory precision preclude evidence accumulation to test the predictions of the traumatic memory in the present. Sets of therapist guided eye movements repeatedly challenge the bias against evidence accumulation and compel sensory sampling of the benign present. Eye movements reset the theta rhythm organizing the flow of information through the brain, facilitating the deployment of both overt and covert attention, and the mnemonic search for associations. Sampling of sensation does not support the predictions of the traumatic memory resulting in prediction error that the brain then attempts to minimize. The net result is a restoration of the integrity of the rhythmic deployment of attention, a recalibration of sensory precision, and the updating (reconsolidation) of the traumatic memory. Thus one prediction of the model is a decrease in Attention Bias Variability, a core dysfunction in PTSD, following successful treatment with EMDR.

## Introduction

The Adaptive Information Processing Model (AIP) guides the development and practice of Eye Movement Desensitization and Reprocessing Therapy (EMDR) used in the treatment of Post-traumatic Stress Disorder. The AIP hypothesizes that “dysfunctionally stored memory” serves as the foundation of post-traumatic psychopathology (Shapiro, 2001). Furthermore “there is a system inherent in all of us that is physiologically geared to process information to a state of mental health… by means of this system, negative emotions are relieved, and learning takes place, is appropriately integrated, and is available for future use” (Shapiro, 2018). EMDR is posited to exert its therapeutic effects through targeted information processing of “dysfunctionally stored memory” (Solomon and Shapiro, 2008; Shapiro and Laliotis, 2011; Shapiro, 2018).

The clinical effectiveness of EMDR has been well-established (Rodenburg et al., 2009; Bisson et al., 2013; Jeffries and Davis, 2013; Watts et al., 2013). However the proposed neurobiological mechanisms of EMDR have yet to offer a model capable of catalyzing robust targeted biological research (Bergmann, 2010). To wit, in a recent review Landin-Romero et al. (2018) concluded, “the current understanding of the mechanisms of action underlying EMDR is similar to the parable of the Blind Men and the Elephant in that there is no agreed definition of what the candidate mechanisms are (i.e., eye movements, bilateral stimulation, dual attention, etc.) and how these mechanisms can be measured or demonstrated.” The goal of this paper is to try to remedy this situation through application of Predictive Processing to EMDR.

## The Free Energy Principle – Foundation of Predictive Processing

Predictive Processing is a corollary of the Free Energy Principle as developed by Friston et al. (2006) and Friston (2009). The Free Energy Principle has its roots in statistical physics as an information isomorph of the second law of thermodynamics (Clark, 2013c). Living requires energy and information to maintain organization and resist the dispersive forces of entropy. For humans this requires statistical processing that minimizes uncertainty about the world, despite not having direct access to the world. As postulated by the Free Energy Principle, this is accomplished through a generative model that is constantly updated to reflect current conditions (Friston et al., 2006). The existence and updating of a generative model is where the Predictive Processing story begins.

## Predictive Processing

The activity of “boot strapping” increasingly complex models of the world based on probabilistic inference is known as Predictive Processing (Clark, 2013c). From the perspective of Predictive Processing the main function of the brain is to predict its own immediate experience, i.e., the patterns of firing neurons that will occur next. To achieve this goal there is a relentless focus on reducing the errors of its predictions so as to “get it right” in the future. This process is known as Prediction Error Minimization and utilizes sensation as feedback on the accuracy of its predictions. The excitement surrounding Predictive Processing in contemporary neuroscience stems from the promise of being able to explain a wide range of cognitive activities including perception, attention, learning, and action with a single conceptually simple mechanism grounded in physiologically plausible computation (Friston, 2009; Hohwy, 2013). Recently this paradigm has been applied to Psychotherapy (Holmes and Nolte, 2019).

## The Predictive Processing Model of EMDR

The Predictive Processing Model of EMDR focuses on the role of memory as the principle substrate for predictions that guide behavior (Bar, 2009; Buckner, 2010). To minimize uncertainty, resist entropy, and ensure survival the brain is constantly making predictions, and then using sensation as feedback to test its predictions (Clark, 2013c). When there is a mismatch between what is predicted and what is currently sensed, the brain registers a “prediction error” (Hohwy, 2013). In response the brain may update the memory through the process of Memory Reconsolidation (Pedreira et al., 2004; Dudai, 2006, 2009). The goal of updating the memory is to minimize the (long-term average of) prediction error thus reducing uncertainty and resulting in more successful behavior in the future. The dysfunctionally stored memories postulated by the AIP make for poor predictions and result in the suboptimal behavior characteristic of PTSD. Thus The Predictive Processing Model of EMDR attempts to explain the biological basis of “the system inherent in all of us that is physiologically geared to process information to a state of mental health” postulated by the AIP and activated by EMDR.

Eye Movement Desensitization and Reprocessing Therapy is an ideal lens through which to view this model as it is “a comprehensive psychotherapy compatible with all theoretical orientations,” and has well-delineated clinical interventions (Shapiro and Laliotis, 2011; Shapiro, 2018). In addition the inclusion of the therapeutic element of eye movements affords the opportunity to appreciate the powerful role that eye movements play in network and mnemonic function (Johansson and Johansson, 2014; Vernet et al., 2014).

## Perception as Inference

Predictive Processing has its roots in the work of German physician and physicist Herman von Helmholtz (Friston et al., 2006). Helmholtz recognized that incoming sensory data are ambiguous (Helmholtz, 1867). For a given sensation there are multiple potential causes in the world. For example, an orange scent could be caused by orange soda, air freshener, or an actual orange. And contrary to common sense, we do not have direct access to the world. Consider vision. Light does not enter the brain. The inside of the skull is dark. Instead the retina converts photons of light into the firing of neurons. In fact every sensory receptor, from vision, to touch, to smell, has the same type of output. This is true for the interoceptive senses such as proprioception, hunger, and thirst as well. In our experience of the world, all the brain has to work with are patterns of firing neurons. As Immanuel Kant suggested, all we can know is the “phenomenon,” that is the effect of the world upon us, i.e., patterns of firing neurons. We can never know “the thing in itself” that is, the actual causes in the world of the effects we experience (Kant, 1781). With this observation Kant anticipated the Markov Blanket, a concept central to Predictive Processing. The Markov blanket is essentially the boundary between a system, and everything else that is not that system, expressed in mathematical terms (Yufik and Friston, 2016). Given the ambiguity of sensory data and the impossibility of knowing “the thing in itself” Helmholtz concluded that perception is an act of unconscious inference. We cannot know directly what lies on the other side of the Markov blanket (i.e., sensory boundary) that is constituted by our sensory epithelia. When we perceive, the brain is making a guess about the state of the world. This process is automatic, rapid, and unconscious (Tenenbaum et al., 2011). As a result we are unaware that a sophisticated process has occurred. We are only aware of the product, what the brain has calculated is the most likely cause, the best guess. However we do not experience this as a probability or a guess, but rather as a fact (Dehaene and Changeux, 2011; Hohwy, 2013) “I see an orange.”

Helmholtz’ hypothesis of perception as inference has significant implications for brain function. If perception is an act of inference, the brain must have information that is used as the basis for inference. That is, it must have a model of the world, *a priori*, before it encounters the world. Dreaming during Rapid Eye Movement (REM) sleep illustrates the ability of the brain to generate perceptual hypotheses in the absence of any sensory data, an *a priori* model (Hobson et al., 2014). In the parlance of predictive processing this is called a prior probability or “prior” based on Bayes Theorem (Geisler and Diehl, 2003). Prior probability is the likelihood of a proposition before considering empirical data from the senses. But where does such a prior probability or model come from?

## Hardwired Models

The models present at birth appear to be hardwired (Ullman et al., 2012). As suggested by Kant, in order for humans to be able to make sense of the world we assume that experience unfolds in extended space, over time, with causes and effects (Kant, 1781). In other words the hardwired model we begin life with contains notions of space, time and causality. Friston has suggested that hardwired models are a function of the type of organism, including its particular sensory receptors and expected environment. Biological systems have a model implicit in their structure, and sample the world so as to fulfill their expectations (Friston et al., 2006). Fish “expect” to be surrounded by water from which they extract oxygen. Humans “expect” to be surrounded by air. Such “proto-concepts” form the scaffolding upon which patterns of firing neurons resulting from experience give rise to more sophisticated models of the world (Ullman, 2019).

## Evolution of Models

Following birth humans “boot strap” increasingly complex models of the world based on experience. Prior probabilities present at birth are modified by experience into posterior probabilities. For example, starting with no knowledge of language, infants learn language. Research is beginning to deconstruct this process through the lens of predictive processing. One of the first tasks of an infant learning language is to parse a stream of syllables into discrete words. Based on patterns of firing neurons from the cochlea, the infant identifies some combinations of sounds as occurring more frequently together than others. Given 2 min of exposure, 8 month old infants can separate the syllables Pre-tty-ba-by into the separate words of pretty, and baby (Saffran et al., 1996). The syllables pre-tty occur more frequently together in natural speech than the syllables found in the middle of the stream, tty-ba. Similarly the syllables ba-by occur more frequently together than tty-ba. The infant’s best guess based on statistical computations about the patterns of firing neurons that it experiences is that “pretty” is a discrete word, and that “baby” is a discrete word. A prior probability that speech sounds that occur in particular patterns have significance, becomes a posterior probability that “pretty” and “baby” are such patterns. This empirically informed “best guess” then becomes incorporated into the infant’s memory and model of language.

## Predictive Processing Implies a Proactive Brain

It is important to underscore that from the contemporary perspective the brain is not simply the passive recipient of sensation that is then used to build a model of the world. To the contrary, the brain is proactive (Raichle, 2010). This is captured in Gregory’s conception of perceptions as hypotheses (Gregory, 1980). From the perspective of predictive processing the brain has a model of the world before it encounters the world. It uses its model to try to predict what it will experience next in its patterns of firing neurons. Action is taken to sample sensation in a manner that tests the hypothesis (Clark, 2013a). To the extent that the prediction about the state of the world is supported by the sampled sensory data, further processing of the sensation is suppressed as it does not contain useful information (Blakemore et al., 2000). If the prediction is not supported, the resulting prediction error will drive further processing of the sensation. In pursuing the brain’s intransigent survival imperative of minimizing prediction error the brain has two main approaches; namely, action and perception (Friston, 2009). With action it can sample the world differently until sampled sensation matches prediction, or it can revise its model. That is, it can update its “prior” to a reality calibrated posterior belief.

## Perceptual Inference-Cycles of Searching the World and Searching Memory

Incoming sensation acts as a retrieval cue for memory (Tulving and Schacter, 1990). For example searching the world with the eyes imports coarse global properties of an object in the form of patterns of firing neurons. Such patterns are believed to trigger an internal hippocampal mediated search that attempts to answer the question “what is this like?” (Bar and Neta, 2008). From this perspective, object recognition is a matching task. An analogy representing the closest familiar representation in memory is selected by the prefrontal cortex from a matrix of possibilities with differing probabilities (Hakonen et al., 2017). Low probability analogies are suppressed (Depue, 2012). The selected analogy is itself connected to a web of associations. Taken together these activated memory networks correspond to the brain’s “best guess” about current reality and what to expect next (Bar, 2009). The brain then tests its prediction by searching the world with saccadic eye movements (Friston et al., 2012). Specifically Friston asserts “…saccadic eye movements are optimal experiments, in which data are gathered to test hypotheses or beliefs…” (Friston, 2012). The data from these eye movements will either support or refute the prediction. If the visual search results do not support the prediction, the brain may attempt to search memory for a new “best guess.” This in turn engenders a new visual search to test the new “best guess.” Searching the world alternates with searching memory in a constant ongoing flux of processing (Richter et al., 2015). This cycle of sampling, matching from memory, prediction, and further sampling continues throughout life as the brain attempts to navigate the endless uncertainty of incoming sensation by minimizing the errors of its predictions (Clark, 2016).

## Eye Movements and Hippocampus Form an Integrated Search System

Perceptual inference as described reflects a process that requires tight coordination between the oculomotor system that controls the movement of the eyes, and the hippocampal search of memory. Converging evidence leads to the conclusion that these two systems are functionally and anatomically coupled (Shen et al., 2016). The nature of this relationship is further illuminated by consideration of the functions of the hippocampus.

## The Hippocampus Navigates Internal and External Space

**FIGURE 1:**
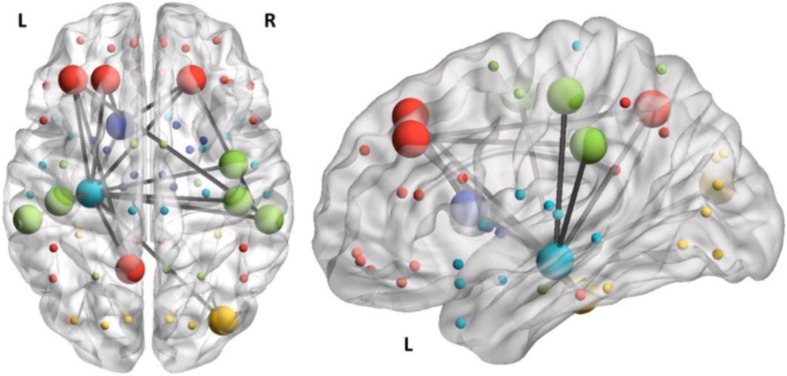
fMRI derived image of successful episodic memory recall showing hippocampal (blue) mediated “retrieval assembly” of cortical regions containing the sensory and motor elements of the memory. Adapted from Geib et al. (2017) and adapted with permission.

The role of the hippocampus in memory function was first described in Scoville and Milner (1957). Subsequently, it was found to play an important role in spatial navigation (O’Keefe and Dostrovsky, 1971). More recently, these two apparently distinct functions have been reconciled through identification of a common underlying mechanism (Buzsaki and Moser, 2013). A leading theory of hippocampal function posits that the hippocampus acts to index the locations in the cortex of the disparate elements of memory which when co-activated confer the experience of remembering (Teyler and Rudy, 2007). In other words the hippocampus knows where in the cortex to find the smell, the sound, the visual image, etc. of an experience allowing reconstruction of an episodic memory (Schacter and Addis, 2007). See Figure 1. In effect the hippocampus maps the physical space inside the brain that gives rise to memories (Bellmund et al., 2018). Similarly the hippocampus maps the disparate landmarks in the physical space outside the brain as it performs its role in navigation in the world. It has been argued that the computational properties of the hippocampus are particularly well-suited to execute this type navigation which is essentially the same whether one is searching the world or searching memory (Buzsaki and Moser, 2013).

## Theta Rhythm Keeps Information Flow Organized

Consider a walk in the park. Crude visual input triggers a hippocampal mediated memory search and retrieval of the best guess regarding current location. Based on the best guess of current location, the brain predicts the next landmark it will encounter using memory (Eichenbaum, 2017). It then tests its prediction by sampling the world with saccadic eye movements (Friston et al., 2012; Parr and Friston, 2017; Stefanics et al., 2018; Smout et al., 2019). In order to execute these cycles the brain must be able to maintain the distinction between new information coming in (encoding) and information already stored in the brain (retrieval). In other words, it must not confuse the monument that is currently seen, with the concession stand that it predicts it will see next based on memory. Recent research suggests that the hippocampal theta rhythm is crucial in organizing the flow of information through the neural circuits responsible for the encoding and retrieval of episodic memory (Hasselmo and Stern, 2014; Siegle and Wilson, 2014).

## Theta Rhythm Correlates With Memory Performance

The theta rhythm has been conceived of as “the navigation rhythm through both physical and mnemonic space, facilitating the formation of maps and episodic/semantic memories” (Buzsaki, 2005). More generally theta rhythms promote coordination across distributed brain areas during different types of information processing (Colgin, 2013). Such coordination of disparate regions including the hippocampus and the prefrontal cortex is critical in being able to retrieve episodic memories (Preston and Eichenbaum, 2013; Geib et al., 2017). Recently, theta rhythm synchronization of hippocampal and prefrontal regions by external stimulation has been shown to transiently restore working memory function in older adults (Reinhart and Nguyen, 2019). The implication is that loss of theta synchronization plays an important role in the deterioration of working memory with age. Given that attention and working memory are the most severely compromised neurocognitive functions in PTSD (Scott et al., 2015), the possibility arises that enhanced theta synchronization might improve memory function in PTSD as well.

## Eye Movements Re-Set Theta Rhythms

The ability to import sensory information is highly dependent on the motor rhythms used to acquire that information (Schroeder et al., 2010). While the eyes are moving during a saccade, visual input to the brain is suppressed (Bremmer et al., 2009). We don’t perceive this because the brain continues to generate its prediction of the world and fills in the gap. If suppression did not occur, our vision would be like a rapidly panning video camera blurring the image every time the eyes moved. In addition such “sensory attenuation” is necessary to keep incoming sensation separated from brain generated prediction (Brown et al., 2013). When the eyes stop moving there is a period of fixation during which data is acquired (Rajkai et al., 2008). It is primarily during fixation that sensation is taken in to the visual system (Wurtz, 2008; Crevecoeur and Kording, 2017). As previously discussed, organizing the incoming and outgoing flow of information through the hippocampus is essential. It appears that saccadic eye movements play a critical role in this regard by resetting the theta rhythm and thus synchronizing the flow of incoming information through disparate regions including the hippocampus and prefrontal cortex in processing experience and memory (Jutras et al., 2013; Meister and Buffalo, 2016).

## Summary of Perceptual Inference

Lacking direct experience of the world perception is an act of inference that utilizes raw sensory data to search memory to find the statistically most likely “best guess” about current reality. The best guess is then tested with saccadic eye movements that sample the world obtaining new data that either support or refute the best guess prediction about the state of the world. The cycling of incoming sensation and outgoing prediction based on memory is perpetual while awake. As part of an integrated search system saccadic eye movements reset the theta rhythm synchronizing the flow of incoming and outgoing information from the hippocampus through other participating structures including the prefrontal cortex. Thus disparate regions are coordinated optimizing the processing of current experience.

## Cross Referencing Sensory Data Reduces Uncertainty

The challenge of perceptual inference about the state of the world becomes more tractable when data from multiple senses is combined into the computations. For example smell offers probabilities of an orange soda, air freshener, or an actual orange. Vision suggests an orange colored ball, or an orange. Touch suggests a tomato, an apple, or an orange. When the statistical probabilities suggested by each sense are integrated, the most likely single cause of these sensations is an orange. In other words cross-referencing by the senses rapidly reduces the possibilities to the most likely cause.

## Memory for Prediction

The clinical relevance of Predictive Processing to psychological trauma and its resolution becomes apparent recognizing that memory is the principle substrate of prediction (Bar, 2009; Buckner, 2010). In fact it can be argued that the raison d’etre of memory at all levels in the brain is to facilitate Predictive Processing (Sterling, 2012). The AIP model postulates “dysfunctionally stored memories” as the foundation of post-traumatic psychopathology. If the core function of the brain is prediction based on memory, it is easy to imagine grossly sub-optimal behavior resulting from such compromised memories. For example when a truck backfires in suburbia, it may trigger a veteran’s dysfunctionally stored memory so he predicts incoming mortar fire and dives to the ground. The subsequent absence of destruction from an incoming mortar represents a massive failure of prediction. If the Predictive Processing account is correct, then the brain would be expected to try to minimize its prediction error to improve future prediction and behavior. The Network Balance Model of Trauma and Resolution postulates that imbalance of the Salience, Default Mode and Central Executive Networks compromises the coordinated interaction of brain regions required to execute this processing (Chamberlin, 2019). However once balance is restored, memory will be processed. But how does memory process? The AIP postulates “there is a system inherent in all of us that is physiologically geared to process information to a state of mental health…by means of this system, negative emotions are relieved, and learning takes place, is appropriately integrated, and is available for future use.” The Predictive Processing Model of EMDR argues that this “inherent system” is, broadly speaking, Predictive Processing itself. In a sense, it is just what the brain does.

## Mismatch Negativity Reflects Prediction Error

Mismatch Negativity is a well-established research paradigm that reflects deviation from the brain’s expectations (Naatanen et al., 2007). When sensation does not match what is expected, the EEG brainwave recorded over the corresponding sensory cortex will show a negative deflection. For example if the 10th note of the song “Mary had a little lamb” is played incorrectly, people familiar with the song will manifest a negative wave in the EEG over the auditory cortex. The brain is surprised. In the parlance of Predictive Processing the brain registers a prediction error. If the song is played incorrectly in the same way multiple times, the magnitude of the measured prediction error will diminish as the brain updates its model and expectations (Baldeweg, 2007; Garrido et al., 2009). In neuro-energetic modeling the magnitude of mismatch negativity has been shown to correlate to the magnitude of prediction error and reflects an increase in energy available to drive synaptic adaptation (Strelnikov, 2007). In effect the brain learns to predict a different pattern of firing neurons (sound) under certain circumstances, e.g., when the song is played by a 5 year-old novice. Prediction error has been minimized. Similar effects have been demonstrated in the visual realm using facial expressions that are unexpected, thus supporting the Predictive Processing paradigm and its postulated updating of models (Stefanics et al., 2018). In the tactile realm, unexpected changes in the intensity of a stimulus result in the updating of somatic models, an effect mediated by the anterior insula (Allen et al., 2015). This has significant implications for the awareness of somatic sensation in trauma, and its processing in EMDR Therapy utilizing bilateral tactile stimuli. It is important to note that mismatch negativity and related violation responses later in peri-stimulus time (e.g., the P 300) are not limited to sensation, but has also been demonstrated on the conceptual levels of grammar and semantic meaning (Naatanen et al., 2007; Batterink and Neville, 2013). Thus Mismatch Negativity appears to reflect the occurrence of prediction error in the brain on multiple levels and in multiple regions.

## Memory Reconsolidation

Following retrieval a memory must undergo a process involving protein synthesis called Memory Reconsolidation in order to return to storage (Nader et al., 2000). This creates an opportunity to alter the memory in several ways. For example pharmacologic interventions may disrupt protein synthesis so the memory cannot return to storage (Przybyslawski et al., 1999; Debiec and Ledoux, 2004; Kindt et al., 2014). Effectively the memory is erased. While promising as a clinical intervention there are many constraints that need to be navigated for the approach to be useful. These constraints are currently the subject of active research (Visser et al., 2018). Another way memory may be altered is physiologically through memory updating or learning. Not only are the causes of sensation uncertain, but the future is inherently uncertain because self and world are constantly changing. This requires that memories be capable of being updated when conditions change in order to optimize predictions (Dudai, 2009; Lee, 2009; Kroes and Fernandez, 2012).

## Memory Reconsolidation Constrained by Boundary Conditions

The process of updating memory is achieved through Memory Reconsolidation and requires that certain conditions be satisfied (Monfils et al., 2009). The first so-called “boundary condition” to be described was a “mismatch between what is expected and what actually occurs” (Pedreira et al., 2004). After a memory is retrieved, if there is a mismatch, i.e., a prediction error, the memory may enter an active state during which new information can be incorporated into the memory. Under these conditions the memory will be updated. However if there is a significant delay in receiving the information that contradicts the expectation, the result will be “extinction,” that is the creation of a new competing memory instead of updating the original memory (Diaz-Mataix et al., 2013). In addition if the new information/experience is too dissimilar to the retrieved memory, a new memory will be created and the retrieved memory will be left intact (Forcato et al., 2009). Thus identifying and controlling boundary conditions are critical if the Memory Updating process is to be harnessed in a therapeutic fashion (Schiller et al., 2010; Kroes et al., 2016; Walker and Stickgold, 2016; Elsey and Kindt, 2017; Treanor et al., 2017).

## Prediction Error Window of Memory Reconsolidation

Several points are worth highlighting regarding memory updating following experience. Memory updating can and does occur spontaneously and without conscious awareness throughout life. And when it occurs, the changes span the range from “intracellular gene inductions to brain-wide systems level reorganization of memory representations” (Stickgold and Walker, 2007). This is consistent with the theoretical formulation of Predictive Processing based on statistical physics that asserts that all quantities that can change in a system will change, in order to minimize prediction error (Friston et al., 2006; Strelnikov, 2010). Furthermore the magnitude of the prediction error appears to be critical in regulating memory updating when attempting a therapeutic intervention. If the prediction error is small, the memory will be not be updated given lack of significant new information. In contrast, if the prediction error is too large, the brain appears to treat it as a new experience and creates a new memory. The original memory is not updated. Only if the prediction error is “moderate” does updating of the memory with new information via reconsolidation occur (Finnie and Nader, 2012; Sevenster et al., 2014; Beckers and Kindt, 2017). In The Predictive Processing Model of EMDR this optimal level of moderate prediction error is referred to as the Prediction Error Window and is a crucial factor in harnessing the therapeutic potential of Memory Reconsolidation.

## Processing Traumatic Experience-Network Balance

It has been postulated that balance of the three principle large-scale networks is an essential pre-requisite for the processing of traumatic experience to an optimal state (Chamberlin, 2019). Such balance may occur spontaneously or through effective trauma therapies such as EMDR. Elements of the EMDR protocol are thought to activate specific individual networks. For example questions during the assessment phase are posited to activate the default mode and salience networks bringing the individual into a state of active re-experiencing. Subsequently therapist guided Dual Attention and Eye Movements are posited to have a crucial role in activating the central executive network thus restoring network balance. This allows the individual to begin taking in new information from the external world and orienting to the present. In essence these interventions set the stage for the “inherent system” postulated by the AIP to then spontaneously process the “dysfunctionally stored memory.” The Predictive Processing Model of EMDR suggests how the “inherent system” may actually function.

## Processing Traumatic Experience-Prediction Error Minimization

Having been brought into a state of active re-experiencing during EMDR, the brain predicts what will come next as the remembered trauma unfolds. For example an individual involved in a car accident predicts the sight of broken glass, the smell of burning plastic, and the pressure of an airbag on the chest. The Predictive Processing Model of EMDR postulates the following sequence of events: saccadic eye movements guided by the therapist compel multi-modal sampling of current sensation thus testing the individual’s predictions of what comes next. Sensory sampling of the therapist’s office does not support the predicted car accident mayhem. There is no broken glass, smell of burning plastic or airbag pressure. The result is multi-modal prediction error. This prediction error registers in the brain as Mismatch Negativity in multiple regions. Energy is mobilized for synaptic adaptation. Memory reconsolidation is initiated. Subsequent sampling is invoked to generate new predictions as the individual attains progressively greater orientation to the benign present. All the while saccadic eye movement mediated theta rhythm synchronization keeps the inflow of sensation and outflow of mnemonic predictions organized for optimal processing. Disparate brain regions are synchronized, and working memory capacity is restored. The net result, the overarching goal of the brain, is prediction error minimization. Ultimately prediction error minimization is driven by the thermodynamics of free energy minimization (Friston, 2010; Sengupta et al., 2013). “There was a car accident but it’s not happening now. It’s over and I’m sitting in an office.”

## Re-Entrant Processing

The preceding example of prediction error minimization occurs over time with repeated sets of subjective reports followed by sets of eye movements. Presumably this involves cycles of re-entrant processing as information gets passed through thalamo-cortical as well as cortico-cortico loops as the processes of disambiguation, differentiation, sensory integration, and mnemonic integration occur (Edelman and Gally, 2013; Preston and Eichenbaum, 2013; Ohkawa et al., 2015; Richter et al., 2015; Hakonen et al., 2017; Kitamura et al., 2017; Yokose et al., 2017; Chao et al., 2018). The result is an updated memory and model of the world that makes better predictions.

## Precision Weighting of Prediction Error

Implicit in the preceding discussion of prediction error minimization is the predictive processing mechanism of precision weighting of prediction error. As noted by Helmholtz incoming sensation is ambiguous. In addition the sensory signal itself it is often imprecise and unreliable. Potentially this sets the stage for an unreliable signal to drive prediction error minimization and memory updating thus compromising the future utility of the brain’s generative model of the world. The predictive processing response to this challenge, postulated to reflect brain function, is to offer a prediction regarding the reliability of the sensory signal. This prediction of reliability is called “precision weighting” and reflects the degree of confidence or precision in the sensory signal (Friston, 2009). It is an estimate of uncertainty that reflects the trustworthiness of the sensation and is posited to be implemented biologically through changes in synaptic gain modulated by “top down” cortical predictions. Signals deemed unreliable and imprecise carry less weight or influence, and are not able to drive learning. In a sense the downward flowing prediction or belief prevails as the interpretation of the current state of the world, and the prediction error based on unreliable sensation is ignored. We then experience what we expect, rather than what sensation might suggest. In contrast, signals deemed reliable have more “weight” with increased synaptic gain, and are able drive memory updating. Thus precision weighting of prediction error can be conceived of as a mechanism for modulating the influence of prediction errors on belief updating (Clark, 2013b). In other words, precision weighting helps us to implicitly ask and answer the following questions: “How much do I trust current sensation? Which sensory channels are the most reliable? And “do I need to update my beliefs?”

## Precision Gives Rise to Attention

Precision weighting also offers a way of understanding sensory attention at a neuronal level. Formally this has been expressed as “attention is the process of optimizing the synaptic gain to represent the precision of sensory information during hierarchical inference” (Feldman and Friston, 2010). This proposition has received strong empirical support in a study of spatial attention and response speed (Vossel et al., 2014). From this perspective attention is an emergent property of the process of estimating the reliability of sensation. As the brain estimates the uncertainty associated with different channels of sensation, giving more weight to some channels through synaptic gain, and less weight to others, the byproduct is what we call “paying attention” (Hohwy, 2013). For example, a sailor proceeding through a dense fog may predict that sound is more reliable than vision, and as a result pays more attention to sound, and relies less on vision than he would on a clear day. Given that the deployment of attention is a crucial factor in the pathology of PTSD, precision weighting may play an important role.

## Attention Compromised in PTSD

A recent meta-analysis found that attention and working memory were among the most severely compromised neurocognitive functions in PTSD (Scott et al., 2015).

Early investigators characterized the abnormalities in attention seen in PTSD as a bias toward threatening stimuli (Fani et al., 2012). While this is frequently demonstrated in clinical populations, there is also a high incidence of bias away from threat, i.e., ignoring threat (Bar-Haim et al., 2010; Sipos et al., 2014). This observation led to the recognition that the abnormalities in attention seen in PTSD are characterized by an increase in Attention Bias Variability (ABV). PTSD sufferers are biased toward the extremes of attention, i.e., excessive attention towards threat at times, and excessive attention away from threat at other times (contributing to reckless behavior) (Iacoviello et al., 2014; Naim et al., 2015). In PTSD the control and deployment of attention appears compromised, thus raising the question of how this might be influenced by precision weighting.

## Precision in Psychological Trauma

Recent empirical work has explored the potential effects of threat on precision weighting in humans (Cornwell et al., 2017). The authors found that under threat of unpredictable aversive shock, there was an increased auditory mismatch response to deviant stimuli best explained by increased post-synaptic gain in primary auditory cortex, with precision weighting biased toward feed forward propagation of prediction errors. This was consistent with a state of anxious hypervigilance and attentional bias to threats in the environment.

Considering the potential role of precision weighting in psychological trauma, Wilkinson et al. (2017) suggested that the survival imperative resulting from experience of a life-threatening event might result in an unusually strong prior probability that will be selected, even when the incoming sensation is a relatively poor fit. The position seems to be, “I must act to ensure survival, evidence be damned.” Recently, this concept has been explored empirically.

Using an agent-based model computer simulation of PTSD (Linson et al., 2019) varied the precision weighting of a prior cued by a stressor and found a perturbation in the balance between exploration and exploitation. Specifically, when the prior was afforded low precision the agent engaged in exploration and evidence accumulation, essentially testing the hypothesis “I’m in danger.” However, when the prior was afforded high precision the agent exploited its knowledge of how to avoid danger and took defensive action, without actually assessing if it was in danger. This was accompanied by physiological responses characteristic of PTSD coded into the model. The authors interpreted these findings by suggesting that a prior belief that carries a high probability of injury or death is afforded high precision via natural selection given that the potential catastrophic consequences outweigh the benefits of exploration and evidence accumulation. (A familiar example of this might be herd behavior when a group of animals run from a predator. While only a subset actually saw the predator, their running triggers in the others the prior of a predator and they take defensive action and start running, without actually trying to see if there is a predator or not. “Better safe than sorry.”).

This suggests that altered precision may result in a state biased against evidence accumulation, consistent with impairments in safety learning characteristic of PTSD (Jovanovic et al., 2012; Sijbrandij et al., 2013). What has been called “safety blindness” (Chamberlin, 2019). And further, that EMDR may act in part by overcoming this bias thus facilitating the acquisition of evidence that does not support traumatic experience in the present. Analysis of the role of eye movements in attention can help illustrate how this might work.

## Attention, Precision, and Eye Movements

What we see depends on where we look. And where we look depends on a guess, a prediction, about where we can find what we are looking for. And what we actually find there, in turn informs where we look next (Parr and Friston, 2017). Thus the motor element of attention, where we look, and the perceptual element, what we see, are mutually informative and interdependent (Mirza et al., 2016). The perceptual and motor elements are part of the perpetual circular processing of the Perception-Action Cycle (Fuster and Bressler, 2015). Elucidating the precise anatomy and physiology of this cycle of visual foraging has been a major challenge for cognitive neuroscience.

The first element involves the motor system and the overt orienting of attention with saccadic eye movements. The second element involves perception and the covert orientation of attention without eye movement. This is the aspect of attention most directly related to precision as previously discussed. This entails orienting to sensation that offers the best evidence in support of the current belief that is being tested (Friston et al., 2012; Mirza et al., 2018). These motor and sensory aspects of attention are tightly coupled sharing a largely overlapping neuroanatomy (Corbetta et al., 1998; Nobre et al., 2000; de Haan et al., 2008). Sharing essentially the same anatomy yet performing dissociable motor and sensory functions (Juan et al., 2008) presents a dilemma that has thus far has defied satisfying explanation. By incorporating neural oscillations the Rhythmic Theory of Attention suggests how this dilemma might be resolved (Fiebelkorn and Kastner, 2019a).

Based on empiric data in humans and monkeys Fiebelkorn and Kaster found rhythmic epochs of enhanced sensory sensitivity alternating with saccadic eye movements during specific phases of theta rhythm. The “sampling state” was characterized by enhanced sensory processing and suppression of attentional shifts, both covert and overt. The “shifting state” was characterized by an attenuation of sensory processing, and was sometimes associated with a covet shift, and sometimes an overt shift in attention. The authors interpreted this to be a state of disengagement that creates an opportunity to shift, either overtly or covertly. These theta “clocked” states were associated with a rhythmic reweighting of network connections to either support motor or sensory activity (Fiebelkorn and Kastner, 2019b). The Rhythmic Theory of Attention posits that the theta rhythm organizes environmental sampling by periodically reweighting functional connections to motor or sensory regions resulting in states that promote either sampling or shifting. Thus the deployment of both overt (saccadic) and covert (precision mediated) attention are tightly coupled, intimately associated with eye movement, and organized by the theta rhythm (“clocking”).

## EMDR May Restore Attention

Taken together these considerations suggest that the therapeutic target of EMDR in PTSD may be in overcoming the bias against exploration and evidence accumulation. Challenging this bias repeatedly with sets of therapist guided eye movements may restore the integrity of the rhythmic deployment of attention (overt and covert) leading to evidence accumulation of a non-traumatic present, recalibration of sensory precision, and the updating of memory. The net result of treatment with EMDR may be relearning how to deploy attention and weigh the sensory evidence we receive from inside and outside the body in support of our narrative about what is happening now. If so, this hypothesis predicts a reduction in ABV and aberrant theta activity following successful treatment with EMDR (Dunkley et al., 2015). Such a reduction would be consistent with recent work utilizing attention control training that resulted in a decrease in PTSD symptoms, ABV (Badura-Brack et al., 2015) and aberrant theta activity (McDermott et al., 2016).

Having described the core elements of the Predictive Processing Model of EMDR it is now possible to posit how some common clinical phenomena from the practice of EMDR might be explained.

## Eyes Move to Remember

Previous discussion of the tight coupling between the oculomotor system and hippocampus elucidated how eye movements can drive search of memory to identify an analogy that matches current sensation thus forming the brain’s best guess. Another manifestation of this integrated oculomotor–hippocampal system is the search of memory that results from eye movements without regard to sensation.

During conversation individuals will periodically look away from the person they are talking to toward regions of the visual field that do not contain any useful information. This so called “Looking at nothing” phenomenon has spawned research that suggests it has an important role in cognition. Also called “non-visual eye movements” or “non-visual gaze paths” the core hypothesis of this research is that saccadic eye movements play a role in non-visual cognitive tasks (Ehrlichman et al., 2007). One finding is that rates of non-visual eye movements increase in tasks requiring search of long term memory and episodic recall (Micic et al., 2010). Going beyond simple association early research found that performance of episodic recall is enhanced with saccadic eye movements (Christman et al., 2003). Subsequent research has established the facilitation of retrieval from memory by eye movements consistent with the concept of embodied cognition (Bochynska and Laeng, 2015; Scholz et al., 2016). The idea is that the specific gaze path traced during the encoding of an experience may enhance recall when it is physically re-enacted during retrieval. Alternatively restriction of eye movement has been shown to impair memory performance (Johansson et al., 2012; Laeng et al., 2014). And finally memory processing during REM sleep is characterized by the elaboration of wide ranging associations while the eyes are closed. These findings suggest that saccadic eye movements have an important role in search and retrieval from memory that is independent of visual input. (The classic Analytic geometry of lying down and staring at the ceiling to facilitate free association appears to support this idea). Indeed it has been suggested that “there is an inherent link, functionally and anatomically between the brain’s oculomotor system and its hippocampal system” (Liu et al., 2016). And further that the physiological coupling between these systems may be obligatory (Andrillon et al., 2015). Taken together these findings suggest the possibility that the operation of the occulomotor–hippocampal system, like many systems in the brain, is bi-directional, and that eye movements may be used deliberately to drive memory search (Christman et al., 2003, 2006; Parker et al., 2008; Brunye et al., 2009; Parker and Dagnall, 2010). While EMDR therapy appears to have incorporated and capitalized on this phenomenon, the explanation offered has been “increased inter-hemispheric brain activity” rather than the bi-directional oculomotor–hippocampal hypothesis advanced above.

## Clearing the Channels of Traumatic Memory

From the preceding discussion it appears that eye movements sometimes occur in the service of vision, e.g., searching the world and sampling reality to test a prediction. In addition eye movements may also occur in the service of memory, e.g., searching memory for associations. Recalling the example of a walk in the park, these distinct roles spontaneously shift rapidly and flexibly throughout waking life. An interesting manifestation of this shifting can be seen during successive sets of eye movements during EMDR. Assessment questions bring the traumatic memory online often engendering a rising level of arousal. Initial sets of eye movements typically result in a rapid “desensitization” with decreasing arousal (Elofsson et al., 2008; Schubert et al., 2011). This appears to result from central executive network activation and amygdala deactivation (de Voogd et al., 2018). The second is increased sampling of current sensation testing the predictions of the traumatic memory (Mirza et al., 2018). This reflects the use of eye movements in the service of vision. When current sensation does not support the predictions of trauma, arousal decreases. However subsequent sets of eye movements are often associated with an increased level of arousal (Sack et al., 2008). Per the Predictive Processing Model this occurs as a result of eye movements in the service of memory. (Seeking uncertainty leads to opportunities to reduce uncertainty). Specifically, eye movements drive the search for associations often finding a new traumatic memory fragment with its corresponding prediction. Returning to the car accident example, the prediction of broken glass is tested by using eye movements and is not supported. Prediction error is minimized and arousal decreases. The next set of eye movements drives memory search and finds the associated fragment of active bleeding from lacerations. This is accompanied by fear and increased arousal. The next set of eye movements then prompts searching the world to test this prediction. The prediction of bleeding is not supported resulting in a fall in arousal. Clinically one result of these cycles of searching the world and then searching memory is an undulating level of arousal with an overall downward trend. The predictions of the traumatic memory, and all its associations are progressively found, tested and not supported. In the words of Friston, “the only hypothesis that can endure over successive saccades is the one that correctly predicts the salient features that are sampled” (Friston, 2012). This leads to an inevitable best guess of current reality: “no trauma happening now.”

## Attention Amplifies Prediction Error

During processing of traumatic experience with EMDR residual symptoms may persist despite significant attenuation. The therapist may then direct the client’s attention to one of the residual symptoms. For example after learning that there is still an abnormal sensation in the abdomen, the therapist may instruct the client to “Go with that” before initiating another set of eye movements. Clinical experience suggests that this intervention is effective in facilitating complete processing of the memory. But how does this work?

Recent research has demonstrated that directing attention to a prediction error amplifies the error signal thus enhancing the neural encoding of the error (Smout et al., 2019). This suggests that the therapist’s directing attention to a residual symptom may amplify the prediction error prompting the brain to minimize the error. That is, the prediction error has been amplified to the “moderate range” where it is in the Prediction Error Window that triggers memory reconsolidation. This appears to result in a complete resolution of the symptom.

## Linking to Adaptive Networks

Another important clinical phenomenon related to eye movement driven elaboration of associations is the linking of the traumatic experience to “adaptive networks” of memory as postulated by the AIP. Network research suggests that elaboration of associations (mental exploration) is the default mode of the brain (Buckner et al., 2008). When the “load” of cognitive and perceptual processing demand is low, the brain searches memory widely (Baror and Bar, 2016). In contrast when the demands are high, the brain utilizes immediate “obvious” information from memory without significant search. For example if there is a gun in your face, your thoughts and associations will probably be very narrowly focused on escape from danger, e.g., door, window. You are unlikely to be reflecting on how guns helped promote survival on the western frontier, or the implications for society of being able to make guns digitally from 3-D printers. The Predictive Processing Model of EMDR postulates that as the prediction error of traumatic memory gets reduced, demand decreases and eye movements drive elaboration of associations progressively more distant from those of the core memory (Christman et al., 2006; Parker et al., 2008, 2009; El Khoury-Malhame et al., 2011). Initially associations will be local, i.e., closely related to, or part of the trauma. From the preceding car accident example, associations might be to bleeding from lacerations, or the ambulance ride to the ER. As demand and arousal decreases associations are broader, and more “global.” For example, “recovering from this car accident was like when I rebounded from the skiing accident. I’m pretty resilient.” This results in a state where there is co-activation of two previously unrelated memories simultaneously. Such synchronous co-activation has been shown to result in formation of a qualitatively new memory that links the previously independent memories (Ohkawa et al., 2015; Yokose et al., 2017). One result is that activation of one memory, e.g., car accident, now triggers activation of the newly linked memories of ski accident and resilience. Per the AIP, the traumatic memory has been linked to an adaptive network.

## Model Predictions

The Predictive Processing Model of EMDR contains multiple predictions that can be empirically tested. For example the model predicts that the processing of traumatic memory with saccadic eye movements in the benign present will result in an increase in prediction error. If so, the increase in prediction error should be reflected by an increase in mismatch negativity. It is also postulated that processing entails serial predictions as associated memory fragments are recalled and tested. If so, the increase in mismatch negativity would be expected to undulate and potentially be synchronized with the undulation of arousal that has been measured. That is an increase in arousal as a new prediction arises, followed by increased mismatch negativity as it is tested and not supported. Over the entire session mismatch negativity (and arousal) would be expected to drop as the memory is updated and the benign present becomes predicted.

The model also suggests that EMDR may act to restore the integrity of the rhythmic deployment of attention including the re-calibration of precision weighting. If so, this would be expected to result in a decrease in aberrant theta dynamics, and a decrease in ABV in patients who experience significant improvement in symptoms.

## Conclusion

The Predictive Processing Model of EMDR builds on The Network Balance Model of Trauma and Resolution utilizing the foundation of the Free Energy Principle to explain how traumatic memories are resolved using EMDR as an example. With the progressive restoration of large-scale network balance, the physiological conditions necessary for the optimal processing of memory are re-established. Next, driven by an excess of Free Energy the brain resumes prediction error minimization of the traumatic memory. Saccadic eye movements facilitate this Predictive Processing resulting in memory updating with reconsolidation and integration into widespread mnemonic networks. EMDR therapy was be used to illustrate how specific clinical interventions may facilitate the processing of “dysfunctionally stored memory” and the resolution of trauma.

## Data Avialability Statement

No new data sets were generated for this review of previously published studies.

## Author Contributions

The author confirms being the sole contributor of this work and has approved it for publication.

## Conflict of Interest

The author declares that the research was conducted in the absence of any commercial or financial relationships that could be construed as a potential conflict of interest.
